# 12/15 lipoxygenase regulation of colorectal tumorigenesis is determined by the relative tumor levels of its metabolite 12-HETE and 13-HODE in animal models

**DOI:** 10.18632/oncotarget.2994

**Published:** 2014-12-18

**Authors:** Jian Chang, Li Jiang, Yinqiu Wang, Bing Yao, Shilin Yang, Bixiang Zhang, Ming-Zhi Zhang

**Affiliations:** ^1^ Department of Medicine, Vanderbilt University School of Medicine, Nashville, TN, USA; ^2^ Cancer Biology, Vanderbilt University School of Medicine, Nashville, TN, USA; ^3^ Hepatic Surgery Center, Tongji Hospital, Tongji Medical College, Huazhong University of Science & Technology, Wuhan, China; ^4^ Department of Biliary and Pancreatic Surgery, Tongji Hospital, Tongji Medical College, Huazhong University of Science & Technology, Wuhan, China; ^5^ Jiangsu Center for the Collaboration and Innovation of Cancer Biotherapy, Cancer Institute, Xuzhou Medical College, Xuzhou, China

**Keywords:** arachidonic acid, linoleic acid, metabolites, epithelial cell, stroma, mice

## Abstract

Colorectal cancer (CRC) continues to be a major cause of morbidity and mortality. The arachidonic acid (AA) pathway and linoleic acid (LA) pathway have been implicated as important contributors to CRC development and growth. Human 15-lipoxygenase 1 (15-LOX-1) converts LA to anti-tumor 13-S-hydroxyoctadecadienoic acid (13-HODE)and 15-LOX-2 converts AA to 15-hydroxyeicosatetraenoic acid (15-HETE). In addition, human 12-LOX metabolizes AA to pro-tumor 12-HETE. In rodents, the function of 12-LOX and 15-LOX-1 and 15-LOX-2 is carried out by a single enzyme, 12/15-LOX. As a result, conflicting conclusions concerning the role of 12-LOX and 15-LOX have been obtained in animal studies. In the present studies, we determined that PD146176, a selective 15-LOX-1 inhibitor, markedly suppressed 13-HODE generation in human colon cancer HCA-7 cells and HCA-7 tumors, in association with increased tumor growth. In contrast, PD146176 treatment led to decreases in 12-HETE generation in mouse colon cancer MC38 cells and MC38 tumors, in association with tumor inhibition. Surprisingly, deletion of host 12/15-LOX alone led to increased MC38 tumor growth, in association with decreased tumor 13-HODE levels, possibly due to inhibition of 12/15-LOX activity in stroma. Therefore, the effect of 12/15-LOX on colorectal tumorigenesis in mouse models could be affected by tumor cell type (human or mouse), relative 12/15 LOX activity in tumor cells and stroma as well as the relative tumor 13-HODE and 12-HETE levels.

## INTRODUCTION

Arachidonic acid (AA), a polyunsaturated omega-6 fatty acid, is a component of the phospholipid domain of most cell membranes[1]. AA is cleaved from membrane phospholipids by cytosolic phospholipase A_2_ and metabolized to eicosanoids by three main pathways: the cyclooxygenase (COX) pathway, the lipoxygenase (LOX) pathway, and the cytochrome P450 pathway. The cyclooxygenase pathway generates prostanoids, including prostaglandins (PGEs) and thromboxane; the lipoxygenase pathway produces leukotrienes and hydroxyeicosatetraenoic acids (HETEs). In the cytochrome P450 pathway, AA is metabolized to epoxyeicosatrienoic acids and HETEs by cytochrome P450 epoxygenases and cytochrome P450 ω-hydroxylases, respectively.

Colorectal cancer (CRC) is one of the most preventable cancers; however it is still the leading cause of cancer death. The arachidonic acid pathway has been implicated as an important contributor to CRC development and growth. Arachidonic acid metabolites derived from the cyclooxygenase, the LOX or the cytochrome P450 pathway have all been implicated in colorectal tumorigenesis[2-4]. COX-derived PGE_2_ is the major prostanoids to promote colorectal tumorigenesis[5]. However, both 5-LOX-derived leukotrienes and 5-HETE have also been implicated in colorectal tumorigenesis[6-10]. Either genetic or pharmacologic inhibition of 5-LOX pathway suppresses colorectal tumorigenesis in murine models[3, 8-11].

In addition to 5-LOX, humans have other two major LOX isoforms: 12-LOX, and 15-LOX. 12-LOX and its metabolite 12-HETE have been reported to promote tumorigenesis and metastasis by inhibiting apoptosis, stimulating cell proliferation, angiogenesis, interactions between tumor cell and vasculature, tumor cell mobility, invasion and proteolysis[12]. Polymorphism (Arg261Gln) in the 12-LOX gene has been associated with a reduced risk of colorectal adenoma[13]. In humans, 15-LOX exists as two isozymes, 15-LOX-1 and 15-LOX-2. The preferred substrate for 15-LOX-1 and 15-LOX-2 are linoleic acid and arachidonic acid, respectively. 15-LOX-1 and its linoleic acid metabolite, 13-HODE (13-S-hydroxyoctadecadienoic acid), are decreased in human colon cancer[14, 15]. 13-S-HODE inhibits cell proliferation and induces apoptosis in colon cancer cells[16].

In rodents, there are not separate 12-LOX and 15-LOX enzymes. Instead, 12/15-LOX in rodents functions as both 12-LOX and 15-LOX. Rodent 12/15-LOX can metabolizes arachidonic acid to 12-HETE and 15-HETE and linoleic acid to 13-HODE. Due to this special property of 12/15-LOX in rodents, information about its role in tumorigenesis in mouse models is limited and controversial. 12/15-LOX has been reported to promote prostate carcinoma progression[17] and to mediate invasion of intrametastatic lymphatic vessels and propagate lymph node metastasis in a mouse model of mammary carcinoma[18]. However, overexpression of 15-LOX-1 in intestinal epithelial cells inhibited colonic tumorigenesis in mice[19]. Using mouse and human colorectal tumor models as well as genetic and pharmacologic inhibition of LOX activity, we determined that the effect of inhibition of LOX activity on colorectal tumorigenesis depends on LOX-derived metabolites in both the tumor cells and the host stroma.

## RESULTS

### Pharmacologic or genetic inhibition of LOX activity had differential effects on murine and human colorectal tumorigenesis

**Figure 1:**
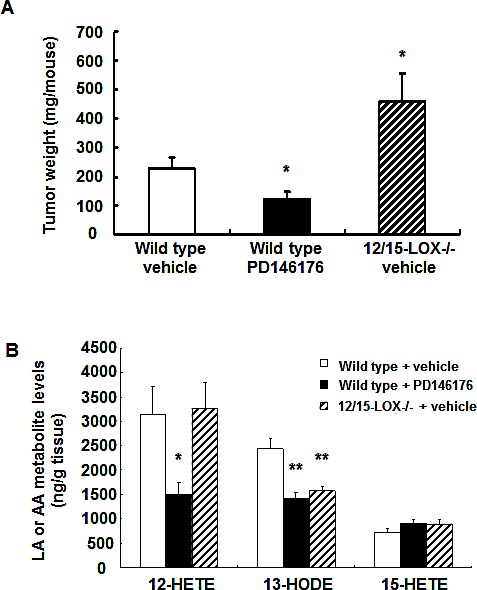
Effects of pharmacologic and genetic inhibition of LOX activity on mouse colonic MC38 tumor growth and tumor eicosanoid production A. Inhibition of LOX activity with PD146176 inhibited, while host 12/15-LOX deletion promoted, MC38 tumor growth (* *P* < 0.05 vs. wild type vehicle, n = 10 in each group). B. PD146176 treatment inhibited MC38 tumor production of both 12-HETE and 13-HODE while host 12/15-LOX deletion only inhibited 13-HODE production (* *P* <0.05 and ** *P* < 0.01 vs. wild type vehicle, n = 4 in each group).

**Figure 2:**
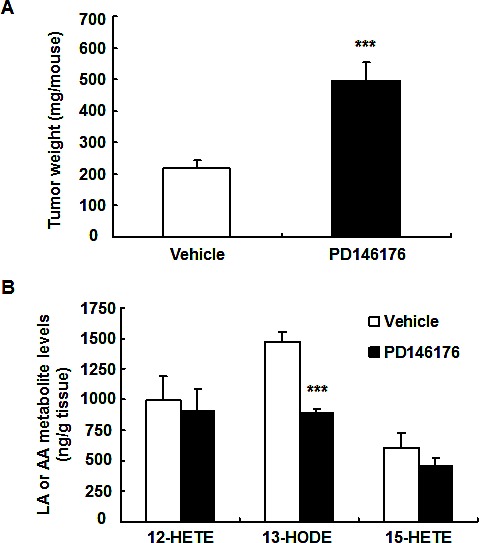
Inhibition of LOX activity with PD146176 led to increased human colonic HCA-7 tumor growth and decreased tumor 13-HODE production A. PD146176 treatment led to increased HCA-7 tumor growth (*** *P* < 0.001 vs. vehicle, n = 6 in each group). B. PD146176 treatment led to decreased 13-HODE production in HCA-7 tumor (*** *P* < 0.001 vs. vehicle, n = 4 in each group).

The effect of 15-LOX on tumorigenesis is limited and controversial[17-19]. To investigate the potential role of 15-LOX in mouse and human colorectal tumorigenesis, mouse colon adenocarcinoma MC38 cells and human colon adenocarcinoma HCA-7 cells were used for mouse and human colonic tumor growth experiments. Treatment with a commercially available 15-LOX-1 inhibitor, PD146176, markedly inhibited mouse MC38 tumor growth (123 ± 24 vs. 227 ± 40 mg/mouse of vehicle, *P* < 0.05, n = 10) (Figure 1A). In contrast, PD146176 treatment promoted human HCA-7 tumor growth (493 ± 62 vs. 217 ± 27 mg/mouse of vehicle, *P* < 0.001, n = 6) (Figure 2A).

Administration of PD146176 inhibits both tumor cell and host 15-LOX activity. To investigate the potential role of host 15-LOX in colorectal tumorigenesis, C57BL/6 wild type or 12/15-LOX knockout mice were subcutaneously injected with MC38 cells and tumor size was evaluated 18 days later. As indicated in Figure 1A, deletion of host 12/15-LOX led to increases in mouse MC38 tumor growth (458 ± 99 vs. 227 ± 40 mg/mouse of vehicle, *P* < 0.05, n = 10), opposite to that seen with PD146176 treatment.

### Eicosanoid profiling in mouse MC38 tumors and human HCA-7 tumors

To investigate the potential mechanisms underlying the differential effects of 15-LOX inhibition on mouse and human colorectal tumorigenesis, we evaluated tumor eicosanoid profiling by gas chromatographic/negative ion chemical ionization mass spectrometric assays using stable isotope dilution[20]. As shown in Figure 1B and supplemental Figure S1A, 12-HETE was the major arachidonic acid metabolite in MC38 tumors, and its production was markedly inhibited by PD146176 treatment (1498 ± 250 vs. 3150 ± 566 ng/gram tissue of vehicle, *P* < 0.05, n = 4), but not by deletion of host 12/15-LOX. No other arachidonic acid-derived metabolites were affected by PD146176 treatment. In addition to arachidonic acid-derived metabolites, MC38 tumors also had high levels of 13-HODE, a linoleic acid-derived metabolite catalyzed by the human 15-LOX-1 enzymatic activity or mouse 12/15-LOX enzymatic activity. The production of 13-HODE in MC38 tumors was markedly inhibited by either PD146176 administration or deletion of host 12/15-LOX (ng/gram tissue: wild type: 2441 ± 212; PD146176: 1412 ± 130, *P* < 0.01; 12/15-LOX deletion: 1577 ± 87, *P* < 0.01. N = 4 in all groups). These results suggest that both 12-HETE and 13-HODE might regulate MC38 tumor growth.

In human HCA-7 tumors, 12-HETE and 13-HODE were the major metabolites of arachidonic acid and linoleic acid (Figure 2B and supplemental Figure S1B). Among all evaluated metabolites from arachidonic acid and linoleic acid in HCA-7 tumors, only the production of 13-HODE was inhibited by PD146176 treatment, suggesting that decreased 13-HODE production might contribute to increased HCA-7 tumor growth in PD146176 treated mice.

### 12/15-LOX enzymatic activity contributed to 13-HODE production in mouse macrophage

**Figure 3:**
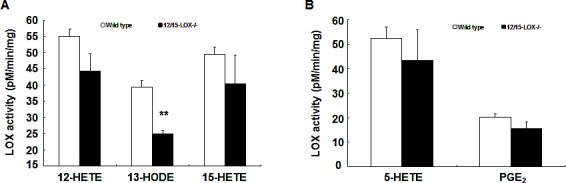
Mouse macrophage 12/15-LOX produced 13-HODE Mouse intestinal macrophages were isolated with CD11b-beads and their abilities to metabolize arachidonic acid and linoleic acid were investigated. 12/15-LOX deletion led to decreased macrophage production of 13-HODE but had no effect on the production of other eicosanoids (** *P* < 0.01 vs. wild type, n = 3 in each group).

In the subcutaneous Xenograft tumors, metabolites of both arachidonic acid and linoleic acid might come from tumor cells or stromal cells (cells from host, including infiltrating immune cells, particularly macrophages). It has been reported that 12/15-LOX is primarily expressed in macrophages in mice[21]. To investigate the potential contribution of macrophages to tumor eicosanoid production, intestinal macrophages from C56BL/6 mice were isolated using CD11b beads, incubated with substrate (60 μM arachidonic acid or 60 μM linoleic acid, 60 min), and metabolites were measured. Enzyme activity was expressed as pM/min/mg protein. As indicated in Figure 3A, macrophages produced similar high levels of 12-HETE, 15-HETE and 15-HODE, all of which could be the products of murine 12/15-LOX. Macrophages also produced 5-HETE and PGE_2_ (Figure 3B). Surprisingly, deletion of 12/15-LOX only significantly inhibited 13-HODE production (pM/min/mg: wild type: 39.3 ± 2.1; 12/15-LOX knockout: 24.9 ± 1.1, *P* < 0.01, n = 3), indicating that macrophage 12/15-LOX has the enzymatic activity converting linoleic acid to 13-HODE.

### PD146176 inhibited 13-HODE production in HCA-7 cells and decreased 12-HETE generation in MC38 cells

**Figure 4:**
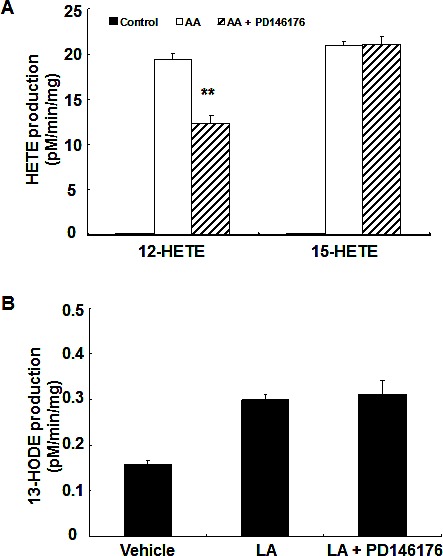
12-HETE production in MC38 cells was inhibited by PD146176 MC38 cells were incubated with arachidonic acid or linoleic acid with or without PD146176, and medium was harvested for measurement of eicosanoid profiling. Enzymatic activity was expressed as pM/min/mg protein. A. PD146176 inhibited the production of arachidonic acid-derived 12-HETE, but not 15-HETE (** *P* < 0.01 vs. vehicle, n = 3 in each group). B. PD146176 had no effect on the production of linoleic acid-derived 13-HODE.

**Figure 5:**
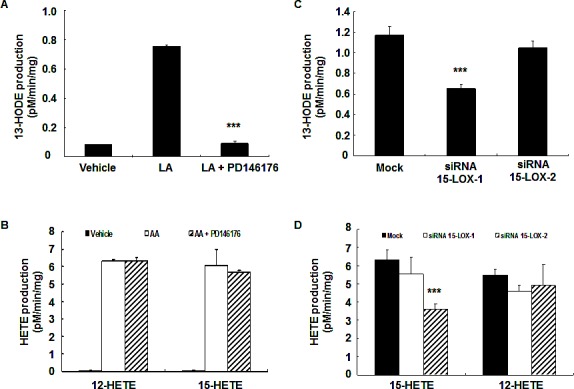
13-HODE production in HCA-7 cells was inhibited by PD146176 HCA-7 cells were incubated with arachidonic acid or linoleic acid with or without PD146176, and medium was harvested for measurement of eicosanoid profiling. Enzymatic activity was expressed as pM/min/mg protein. A. PD146176 markedly inhibited the production of linoleic acid-derived 13-HODE production (*** *P* < 0.001 vs. vehicle, n = 3 in each group). B. PD146176 had no effect on the production of arachidonic acid-derived 12-HETE and 15-HETE production. C. Gene silence of 15-LOX-1 markedly inhibited the production of linoleic acid-derived 13-HODE production (*** *P* < 0.001 vs. vehicle, n = 3 in each group). D. Gene silence of 15-LOX-2 markedly inhibited the production of arachidonic acid-derived 15-HETE (*** *P* < 0.001 vs. vehicle, n = 3 in each group).

To investigate the effect of PD146176 on the production of metabolites from arachidonic acid or linoleic acid, MC38 cells and HCA-7 cells were incubated with substrate (arachidonic acid or linoleic acid) with or without the presence of PD146176. As indicated in Figure 4, PD146176 inhibited 12-HETE production in MC38 cells (12.37 ± 0.82 vs.19.45 ± 0.60 pM/min/mg of vehicle, *P* < 0.01, n = 3), but had no effect on the production of 15-HETE and 13-HODE. In contrast, PD146176 inhibited 13-HODE production in HCA-7 cells (0.09 ± 0.02 vs. 0.75 ± 0.01 pM/min/mg of vehicle, *P* < 0.001, n = 3), but had no effect on the production of 12-HETE and 15-HETE (Figure 5 A&B).

To further investigate the eicosanoid profiling in mouse MC38 cells metabolized by 12/15-LOX and in human HCA-7 cells by 12-LOX and 15-LOX-1 and 15-LOX-2, siRNA technique was used to silence these genes. In MC38 cells, gene silence of Alox15 (12/15-LOX) significantly inhibited the production of 12-HETE (15.27 ± 1.13 vs. 26.75 ± 2.26 pM/min/mg of mock control, *P* < 0.001, n = 3), but had no effect on the production of 13-HODE (0.414 ± 0.022 vs. 0.381 ± 0.041 pM/min/mg of mock control, *P* > 0.05, n = 3) and 15-HETE (23.74 ± 3.13 vs. 25.43 ± 2.64 pM/min/mg of mock control, *P* > 0.05, n = 3). In human HCA-7 cells, gene silence of 15-LOX-1 but not 15-LOX-2 inhibited the production of LA-derived 13-HODE (0.652 ± 0.040 vs. 1.171 ± 0.088 pM/min/mg of mock control, *P* < 0.001, n = 3), while gene silence of 15-LOX-2 but not 15-LOX-1 inhibited the production of AA-derived 15-HETE (3.620 ± 0.285 vs. 6.326 ± 0.537 pM/min/mg of Mock control, *P* < 0.001, n = 3) (Figure 5 C&D). Gene silence of 12-LOX in HCA-7 cells inhibited the production of AA-derived 12-HETE (3.583 ± 0.279 vs. 5.477 ± 0.322 pM/min/mg of Mock control, *P* < 0.001, n = 3).

### 13-HODE inhibited and 12-HETE stimulated both murine and human colon cancer cell proliferation

**Figure 6:**
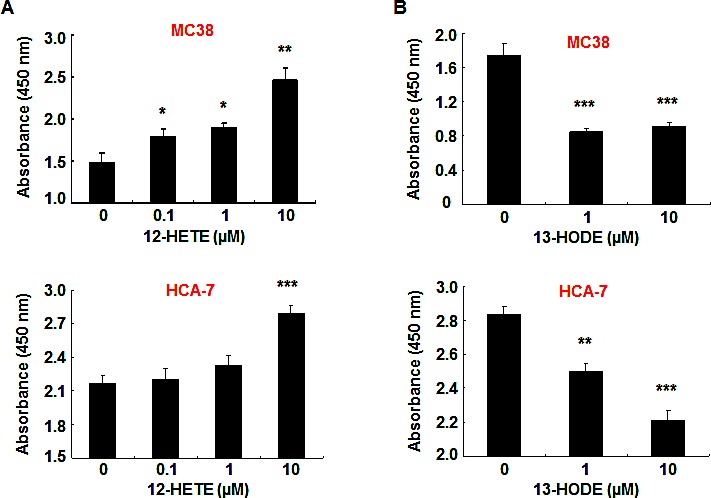
12-HETE stimulated and 13-HODE inhibited murine and human colonic cancer cell proliferation A. 12-HETE stimulated both murine MC38 and human HCA-7 colon cancer cell proliferation (* *P* <0.05, ** *P* < 0.01, *** *P* < 0.01. N = 4). B. 13-HODE inhibited both murine MC38 and human HCA-7 colon cancer cell proliferation (** *P* < 0.01, *** *P* < 0.01. N = 4 in HCA-7 cells and n = 6 in MC38 cells).

To investigate the direct effects of 12-HETE, 15-HETE and 13-HODE on colon cancer proliferation, we treated different mouse and human colon cancer cell lines with these agents and cell proliferation was measured by using cell proliferation assay kit. As shown in Figure 6A, 12-HETE stimulated MC38 cell proliferation at 0.1 μM, but stimulated HCA-7 cell proliferation at 10 μM, indicating that 12-HETE is a more potent mitogen for MC38 cells than HCA-7 cells. In addition, 12-HETE also potently stimulated the proliferation of CT26 cells, another mouse colon cancer cell line (supplemental Figure S2). On the other hand, 13-HODE inhibited the proliferation of all tested mouse and human colon cancer cell lines (Figure 6 and supplemental Figure S2). 15-HETE had no effect on cell proliferation in all cell lines investigated (data not shown).

**Figure 7:**
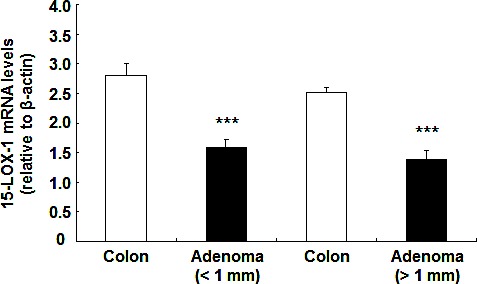
15-LOX-1 mRNA levels were decreased in human colonic adenomas Human colonic adenomas and paired normal colonic tissues were used for qPCR. 15-LOX-1 mRNA levels were decreased in both small (<1cm) and larger (>1cm) adenomas compared to paired normal colonic tissues (*** P < 0.001, n = 7).

Human 15-LOX-1 mRNA levels were decreased in human colonic adenomas. Shureiqi et al reported that 13-HODE levels in human colorectal cancer mucosa and polyp mucosa were reduced compared to paired normal colon, while 12-HETE, 15-HETE and leukotriene B4 levels were comparable between normal colon and colorectal tumors[15]. Therefore we evaluate 15-LOX-1 mRNA levels between adenomas with different sizes and their paired normal colon. As indicated in Figure 7, 15-LOX-1 mRNA levels were significantly reduced in both small and larger adenomas compared with normal colons (***P < 0.001 vs. paired normal colon, n = 7 in each group).

## DISCUSSION

**Figure 8:**
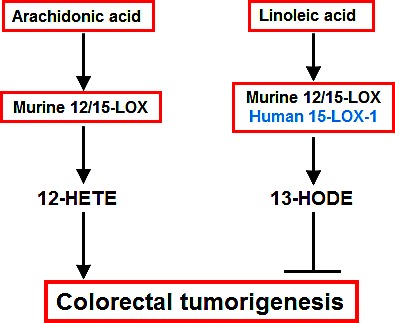
Proposed schema for arachidonic acid and linoleic acid metabolism-mediated colorectal tumorigenesis Both rodent 12/15-LOX and human 15-LOX-1 convert linoleic acid to 13-HODE to inhibit colorectal tumorigenesis. In addition, rodent 12/15-LOX can also convert arachidonic acid to 12-HETE to promote colorectal tumorigenesis. The potential role of 12/15-LOX in murine colonic cancer models should consider tumor cell type (human or mouse), the relative 12/15-LOX activity in tumor cells and stroma, and the generation of 12-HETE and 13-HODE in the tumors.

The current studies were undertaken to investigate the potential role of 15-LOX in colorectal tumorigenesis. The major findings include the following: (1) administration of PD146176, a commercially available, potent and selective inhibitor of 15-LOX-1, promoted human colonic HCA-7 tumor growth in association with decreased tumor levels of 13-HODE, which is consistent with the observation that human 15-LOX-1 suppresses the development and growth of human colorectal cancer; (2) PD146176 treatment inhibited murine colonic MC38 tumor growth in association with decreased tumor levels of both 12-HETE and 13-HODE; (3) 12/15-LOX deletion in the host tissue promoted MC38 tumor growth in association with decreased tumor 13-HODE alone; (4) PD146176 inhibited 13-HODE production in HCA-7 cells but inhibited 12-HETE production in MC38 cells; (5) 12/15-LOX deletion led to decreased production of 13-HODE in isolated intestinal macrophages; and (5) 12-HETE stimulated and 13-HODE inhibited both murine and human colon cancer cell proliferation. Taken together, these results suggest that PD146176 might promote human colorectal tumor growth primarily due to inhibition of 13-HODE production, but inhibit murine colorectal tumor growth as a result of inhibition of 12-HETE generation. The proposed mechanisms underlying rodent 12/15-LOX regulation of colorectal tumorigenesis are summarized in Figure 8.

Three murine 12/15-LOX isoforms exist: the leukocyte-type, the platelet-type, and the epidermis-type. The leukocyte-type 12/15-LOX is the major isoform and possesses high enzymatic activity to generate 12-HETE and 15-HETE from arachidonic acid and 13-HODE from linoleic acid[22]. The leukocyte-type 12/15-LOX is primarily expressed in macrophages, and mice with deletion of this isoform were used in our current studies[21, 23].

In a xenograft tumor model, tumors contain epithelial cells from exogenously injected cells and stroma cells from host. Tumor-stromal interactions play a key role in the development and progression of cancers. The solid tumor stromal microenvironment consists of infiltrating immune cells, fibroblasts, myofibroblasts, adipocytes, endothelial cells and pericytes as well as a variety of extracellular matrix components[24-26]. Macrophages are a major component of stroma and have been reported to play an important role in colorectal tumorigenesis. Macrophages may release various chemicals and cytokines and chemokines that regulate tumor cell proliferation or apoptosis via paracrine action. In the current studies, we found that 12/15-LOX deletion led to marked decreases in the production of 13-HODE in mouse intestinal macrophages, suggesting that mouse macrophage 12/15-LOX can convert linoleic acid to 13-HODE. As PD146176 also inhibited the production of 13-HODE in human colon cancer HCA-7 cells, we postulate that PD146176-induced decreases in 13-HODE levels in HCA-7 tumors were due to inhibition of human HCA-7 tumor cell 15-LOX-1 activity and in mouse MC38 tumors, were due to inhibition of 12/15-LOX activity (particularly in macrophages).

In the cultured mouse colon cancer MC38 cells, PD146176 treatment inhibited the production of 12-HETE but not the production of 13-HDOE and 15-HETE. 12/15-LOX deletion had no effect on the production of 12-HETE and 15-HETE in isolated mouse macrophages. Therefore, we suggest that the tumor promoting effect of host 12/15-LOX deletion was due to inhibition of production of stromal cell 12/15-LOX-derived 13-HODE (Figure 6). On the other hand, our results indicate that PD146176 inhibited MC38 tumor growth due to inhibition of MC38 cell 12/15-LOX-derived 12-HETE, which is a potent mitogenic agent for MC38 cells (Figure 6).

Epidemiological studies in humans have suggested that high intake of linoleic acid can protect against tumorigenesis[27]. Both human 15-LOX-1 and rodent 12/15-LOX utilize linoleic acid as substrate to generate tumor suppressive 13-HODE. Peroxisome proliferator-activated receptors (PPARs) are transcription factors that strongly influence molecular events in normal and cancer cells. PPARs can act as nuclear receptors for 13-HODE. PPAR-δ expression promotes colonic tumorigenesis[28], while activation of PPARγ inhibits cell proliferation and induces apoptosis in a retinoblastoma protein-dependent manner[29]. 13-HODE has been reported to inhibit the proliferation of colorectal cancer cells through down-regulation of PPAR-δ signaling and promotion of PPAR-γ activity[16, 30]. Linoleic acid is a major component of fat in commercially available rodent diet. For example, Basal Diet (Catalog # 7024, TestDiet, St. Louis, MO) contains 3.34% linoleic acid, the major component of fat in the diet. Therefore, there is enough substrate for 12/15 LOX to generate 13-HODE *in vivo*.

Although investigation of the role of 12/15-LOX in colorectal tumorigenesis is limited and controversial in animal models[18, 19], studies in human colorectal tumorigenesis have consistently indicated that15-LOX-1 and its linoleic acid metabolite, 13-HODE, inhibit tumor growth[14, 16, 31]. 13-HODE inhibits human colorectal carcinoma cell proliferation and overexpression of 15-LOX-1 inhibits human colon cancer cell proliferation and induces apoptosis[32-34]. Shureiqi et al. recently reported that 13-HODE levels were decreased in colorectal polyp mucosa and in colorectal cancer mucosa compared with paired normal colon, while the levels of other lipoxygenase-mediated metabolites including 12- or 15-hydroxyeicosatetraenoic acid (12-HETE, 15-HETE) or leukotriene B4 levels were comparable between normal colon and colorectal tumors[15]. In addition, induction of 15-LOX-1 expression and 13-HODE production has been reported to contribute to colorectal cancer cell apoptosis induced by NSAIDs[35]. Even expression of human 15-LOX-1 in mouse colonic epithelial cells can suppress colonic tumorigenesis[19]. Although eicosanoid profiling was not available in this study, it is possible that 13-HODE production increased in these cells, because linoleic acid, the substrate of human 15-LOX-1, is robust in rodent food as described above.

In summary, 15-LOX-1 and its linoleic acid metabolite, 13-HODE, inhibit human colorectal tumorigenesis. However, the effect of rodent 12/15 LOX on colorectal tumorigenesis is complicated and is determined by several factors: tumor cell type (human or mouse), tumor cell 12/15 LOX level, host 12/15-LOX activity, relative production of arachidonic acid and linoleic acid metabolites, and availability of substrate.

## METHODS

### Ethics Statement

Investigation has been conducted in accordance with the ethical standards and according to the Declaration of Helsinki and according to national and international guidelines and has been approved by the Institutional Animal Care and Use Committee of Vanderbilt University.

### Animals

12/15-LOX knockout mice (C57BL/6, stock number 002778) were purchased from Jackson Laboratories. Athymic nude mice were purchased from Harlan Sprague Dawley (male, 8 week old).

### Cell culture and cell proliferation assays

Mouse colon adenocarcinoma MC38 cells (C57BL/6) and CT26 cells (BALB/c), human colon carcinoma HCA-7 cells, KM12 cells and DLD cells were grown in RPMI-1640 supplemented with 4,500 mg/L glucose, 2 mM L-glutamine, 10% fetal bovine serum, 100 U/ml penicillin, and 100 μg/ml streptomycin in 5% CO_2_ and 95% air at 37^o^C[36]. The cells were seeded in a 96-well plate (1 × 10^4^ cells in 200 μl medium) for 16 hours, then starved for 24 hours in medium containing 0.1% fetal bovine serum, and various concentrations of agents (12-HETE, 15-HETE and 13-HODE) were added for additional 4 hours. Then BrdU solution was added to each well for 4 hours. Analysis was performed according to the manufacturer's protocol (BrdU cell proliferation assay kit, Cat#6813, Cell Signaling).

### Primary tumor growth

For mouse colon adenocarcinoma MC38 tumor experiments, male wild type and 12/15-LOX−/− mice (C57BL/6, 8 weeks old) were given 2 dorsal s.c. injections of MC38 cells (5 × 10^5^ cells per site). Wild type mice were treated with vehicle (water) or 12/15-LOX inhibitor, PD146176 (5 mg/kg/day, mini-pump, 2004) and sacrificed after 18 day of growth. Tumors were weighed at the end of experiment. For human colon carcinoma experiments, athymic nude mice were given 2 dorsal s.c. injections of HCA-7 cells (3.35 × 10^6^ cells per site) with administration of vehicle (water) or PD146176 (5 mg/kg/day, mini-pump, 2004) and sacrificed after 4 week of growth. Under anesthesia with Nembutal (60 mg/kg, i.p.), the tumor was dissected, weighed, and snap-frozen in liquid N_2_ for eicosanoid measurement and other analysis or fixed in FPAS for immunohistochemistry[37].

### Isolation of intestinal monocytes/macrophages

Intestinal monocytes/macrophages in single–cell suspensions were enriched using mouse CD11b Microbeads and MACS columns (Miltenyi Biotec) following the manufacturer's protocol.

### Enzyme activity assays

Either isolated monocytes/macrophages or cultured cells were incubated in serum-free RPMI-1640 medium containing 60 μM arachidonic acid or linoleic acid for 1 hour, medium was harvested for eicosanoid measurements. Protein concentration was determined by using Pierce BCA protein assay kit (Cat#23225, Thermo Scientific). Enzyme activity was expressed as pM/min/mg protein.

### RNA interference

siRNAs and negative control siRNA (mock) included ALOX12-human (Cat# 4392420), ALOX15-human (15-LOX-1, cat#4392420), ALOX15B-human (15-LOX-2, cat# 4392420), Alox15-mouse (12/15-LOX, cat# 4390771) and Silencer Selective Control NO.1 siRNA (cat# 4390843) were purchased from Invitrogen. Cells were seeded at a density of 3×10^5^ cells per well on 6-well plates and grown in 10% FBS/DMEM without antibiotics. After 24 h, the cells were transfected with siRNA using Lipofectamine® RNAiMAX (Invitrogen) according to the manufacturer's instruction. Briefly, 25 pmol siRNA and 7.5 μl RNAiMAX (Invitrogen) were diluted in Opti-MEM (Invitrogen) without serum and antibiotics. After mixing for 15 min at room temperature, the mixture was added to a final volume of 1.5ml, and the cells were incubated at 37 °C in a humidified incubator. After 24 h incubation, the interference mixture was removed, and complete culture medium was added. The enzyme activity assays were performed in the cells at 72 h after siRNA transfection as described above.

### Measurement of eicosanoids

Tumor tissue and culture medium eicosanoid profiling was measured by gas chromatographic/negative ion chemical ionization mass spectrometric assays using stable isotope dilution, as described previously[20, 38].

### RNA isolation and quantitative real-time PCR

Total RNA was isolated from tumors using TRIzol reagents (Invitrogen) according to the manufacturer's instructions. Quantitative PCR was performed using TaqMan real time PCR machine (7900HT, Applied Biosystems). The Master Mix and gene probes were also purchased from Applied Biosystems. The primers used for human ALOX15 (Hs00993766) and β-actin (Hs99999903) were from Applied Biosystems.

### Huuman tissues

The human samples used in this study were from the Tennessee Colorectal Polyp Study (TCPS) protocol that was approved by the Institutional Review Board of Vanderbilt Medical Center, and written, informed consent was obtained from all participants.

### Statistics

All values are presented as means, with error bars representing ± s.e.m. ANOVA and Bonferroni *t* tests were used for statistical analysis, and differences were considered significant when *P*<0.05.

## SUPPLEMENTARY MATERIAL, FIGURES, TABLES


