# Supplementary material for: 12/15 lipoxygenase regulation of colorectal tumorigenesis is determined by the relative tumor levels of its metabolite 12-HETE and 13-HODE in animal models
Source: Oncotarget. 2014 Dec 18;6(5):2879–88. doi: 10.18632/oncotarget.2994 (PMC4413624; doi:10.18632/oncotarget.2994)
Supplement: Supplementary file 1 [file oncotarget-06-2879-s001.pdf]

12/15 lipoxygenase regulation of colorectal tumorigenesis is determined by the relative tumor levels of its metabolite 12-HETE and 13-HODE in animal models

Supplementary Material

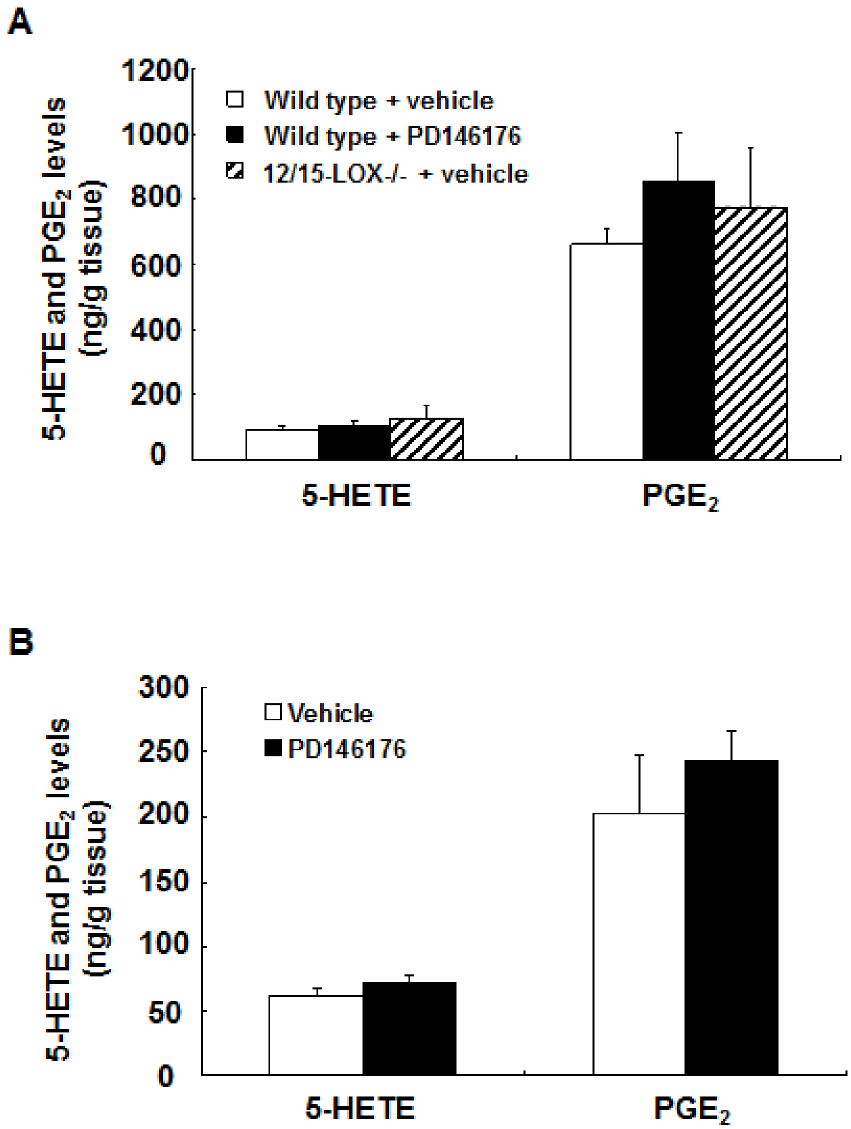

Figure S1: PD146176 treatment and host 12/15-LOX deletion had no effect on the production of 5-HETE and PGE_2 in colonic tumors. **A**: Either PD146176 treatment or host 12/15-LOX deletion had no effect on MC38 tumor levels of 5-HETE and PGE_2 (N = 4 in each group). **B**: PD146176 treatment had no effect on HCA-7 tumor levels of 5-HETE and PGE_2 (N = 4 in each group).

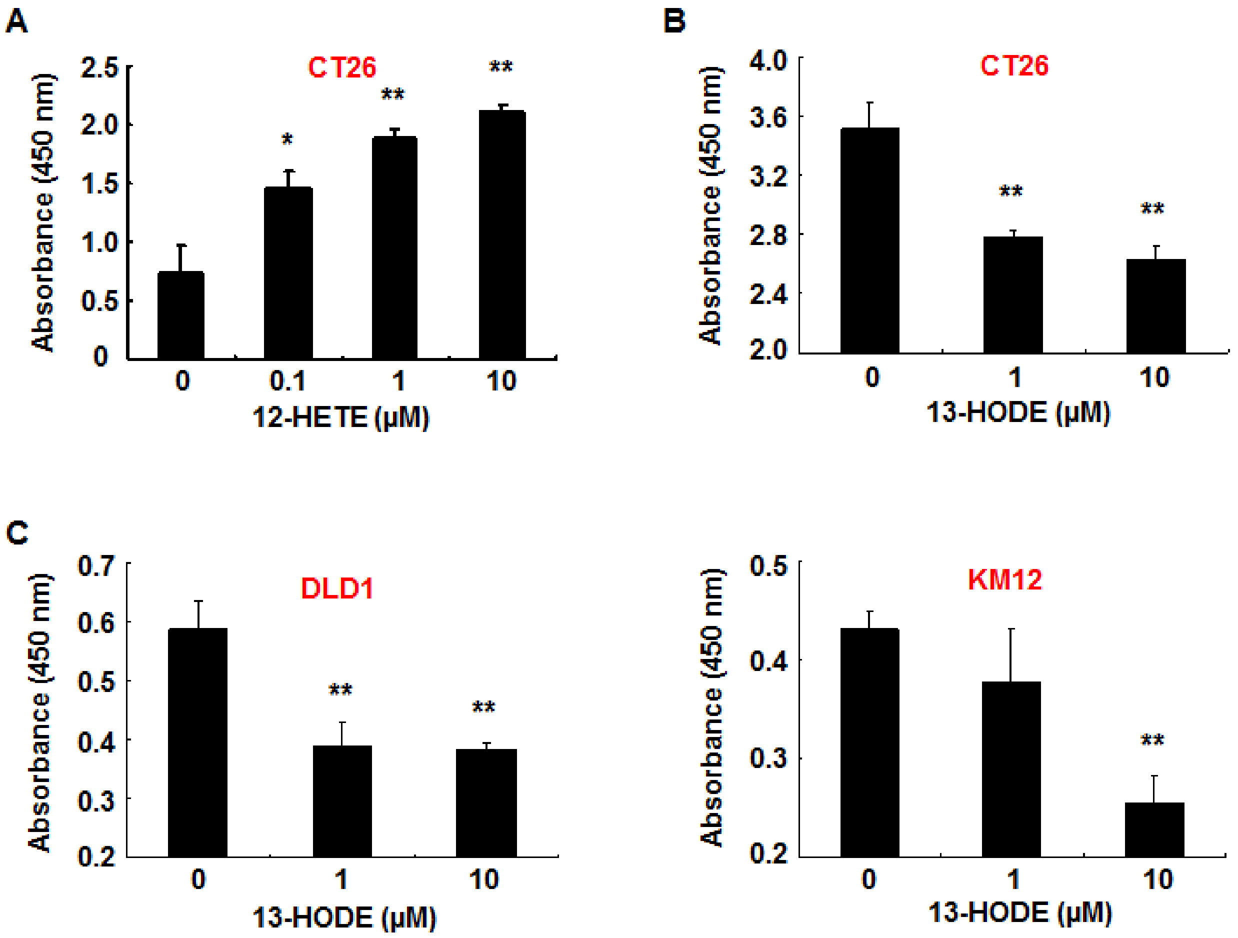

Figure S2: 12-HETE stimulated and 13-HODE inhibited murine and human colon cancer cell proliferation. A. 12-HETE stimulated murine CT26 colon cancer cell proliferation (* *P* <0.05, ** *P* < 0.01. N = 4). **B**. 13-HODE inhibited murine CT26 colon cancer cell proliferation (** *P* < 0.01. N = 4). **C**. 13-HODE inhibited both human DLD and KM12 colon cancer cell proliferation (** *P* < 0.01. N = 4 in KM12 cells and n = 6 in DLD1 cells).
